# Phylogenomics shows unique traits in Noctilucales are derived rather than ancestral

**DOI:** 10.1093/pnasnexus/pgac202

**Published:** 2022-09-22

**Authors:** Elizabeth C Cooney, Brian S Leander, Patrick J Keeling

**Affiliations:** Department of Botany, University of British Columbia, British Columbia, Vancouver V6T 1Z4, Canada; Department of Botany, University of British Columbia, British Columbia, Vancouver V6T 1Z4, Canada; Department of Zoology, University of British Columbia, British Columbia, Vancouver V6T 1Z4, Canada; Department of Botany, University of British Columbia, British Columbia, Vancouver V6T 1Z4, Canada

**Keywords:** Noctilucales, dinoflagellate evolution, Amphidinium, Abediniales, single cell transcriptomics

## Abstract

Dinoflagellates are a diverse protist group possessing many unique traits. These include (but are not limited to) expansive genomes packaged into permanently condensed chromosomes, photosynthetic or cryptic plastids acquired vertically or horizontally in serial endosymbioses, and a ruffle-like transverse flagellum attached along its length to the cell. When reconstructing character evolution, early branching lineages with unusual features that distinguish them from the rest of the group have proven useful for inferring ancestral states. The Noctilucales are one such lineage, possessing relaxed chromosomes in some life stages and a trailing, thread-like transverse flagellum. However, most of the cellular and molecular data for the entire group come from a single cultured species, *Noctiluca scintillans*, and because its phylogenetic position is unresolved it remains unclear if these traits are ancestral or derived. Here, we use single cell transcriptomics to characterize three diverse Noctilucales genera: *Spatulodinium, Kofoidinium*, and a new lineage, *Fabadinium* gen. nov. We also provide transcriptomes for undescribed species in *Amphidinium* and Abediniales, critical taxa for clarifying the phylogenetic position of Noctilucales. Phylogenomic analyses suggests that the Noctilucales are sister to *Amphidinium* rather than an independent branch outside the core dinoflagellates. This topology is consistent with observations of shared characteristics between some members of Noctilucales and *Amphidinium* and provides the most compelling evidence to date that the unusual traits within this group are derived rather than ancestral. We also confirm that *Spatulodinium* plastids are photosynthetic and of ancestral origin, and show that all non-photosynthetic Noctilucales retain plastid genes indicating a cryptic organelle.

Significance Statement The Noctilucales are an early branching dinoflagellate lineage with peculiar traits thought to reflect those of the early ancestor of the core group. Little is known about the diverse members of the Noctilucales or its phylogenetic position since comprehensive molecular data is only available for one species. Since the rest of the Noctilucales are not available in culture, we sequenced transcriptomes from single cells collected from the field, expanding transcriptome data for Noctilucales to include three more members, including a new genus. These data reveal that the atypical biology of Noctilucales probably does not reflect that of an early dinoflagellate ancestor as previously thought, but is rather more recently derived within the group.

## Introduction

Dinoflagellates are a diverse and abundant group of protists that play many important and complex roles in aquatic environments worldwide (1, 2). Approximately half of known species are photosynthetic, some forming symbioses (3, 4), and many create massive blooms, contributing substantially to coastal primary production (5). Blooms of toxic species sometimes threaten fisheries and human health (6); however, many heterotrophic and mixotrophic dinoflagellates are prolific grazers that help diminish such blooms (7, 8, 9). Additionally, parasitic taxa contribute to the microbial loop by infecting and killing their bloom-forming counterparts and other members of the planktonic community (10, 11).

In addition to their ecological relevance, dinoflagellates have many unusual traits that have expanded our understanding of eukaryotic cell biology. Perhaps best known for their unique nuclei, dinoflagellates have massive genomes (up to 245 Gb of DNA in some cases) characterized by many tandem arrays of duplicate genes that require trans-splicing for expression (12, 13, 14). Although the mechanism is still poorly understood, chromatin packaging in dinoflagellates is non-nucleosomal, driven by horizontally acquired bacterial histone-like proteins (HLPs), and dinoflagellate-viral nucleoproteins (DVNPs) instead of canonical histones (15, 16, 17). The resulting liquid crystalline chromosomes, which give the dinoflagellate nucleus (or “dinokaryon”) its distinctive fibrillar appearance, remain condensed throughout the cell cycle (18). Another area of interest is the history and nature of plastid evolution, which is characterized by secondary and tertiary plastid acquisition (19) and frequent independent losses of photosynthesis that leave behind a cryptic plastid organelle with reduced functions (20, 21).

Reconstructing ancestral states can be greatly informed by characterizing deep-branching sister groups to the lineage in question, which has been used frequently in understanding the evolution of dinoflagellates and their close relatives, the Apicomplexa (22–24, 25). One taxon with recognized potential to clarify dinoflagellate evolution is *Noctiluca scintillans*, which is thought to be a deep branch in the dinoflagellate lineage and possesses aberrant forms of some important dinoflagellate traits. For example, *Noctiluca* chromosomes are only condensed during sporogenesis, becoming relaxed in the trophont stage (26). Together with their presumed deep-branching position, this suggests that the dinokaryon first arose as a transient feature before eventually becoming permanent, and that other unique characteristics of this group might also be ancestral. However, the strength of such conclusions is undermined by the fact that most morphological data and nearly all molecular data for all Noctilucales comes from the only species in culture, *N. scintillans*. Observations of other large, hyaline dinoflagellates, and more recent small subunit ribosomal RNA (SSU) gene surveys, have both shown that many other noctilucoid dinoflagellates exist globally, but are rare (27–30, 31, 32). While some taxa have bulbous morphologies reminiscent of *N. scintillans* (33, 34, 35), the Leptodiscaceans tend to be flat with strong bilateral symmetry (36). Recently, transcriptome data revealed that one presumptive Leptodiscaceae genus, *Abedinium*, actually branches sister to the core dinoflagellates and Noctilucales collectively (37). In general, data from the group are so sparse that interpretation of their character states remains uncertain.

In this study, we expand the available transcriptome data for the Noctilucales to include three more genera: *Spatulodinium, Kofoidinium*, and a new lineage we name *Fabadinium* gen. nov, as well as transcriptomes from other key taxa, *Amphidinium* and an undescribed lineage within Abediniales. With these data, we identified the distribution of genes relating to plastid function, and also carried out a robust phylogenomic analysis that resolves the relationships within the Noctilucales and their relationship to core dinoflagellates. Most importantly, we show that the Noctilucales are not a sister to core dinoflagellates, but more likely sister to *Amphidinium*. Our analysis reveals that many of the traits distinguishing the Noctilucales from other dinoflagellates are derived rather than ancestral.

## Results and discussion

### Identification and classification of noctilucoids and relatives

**Fig. 1.**
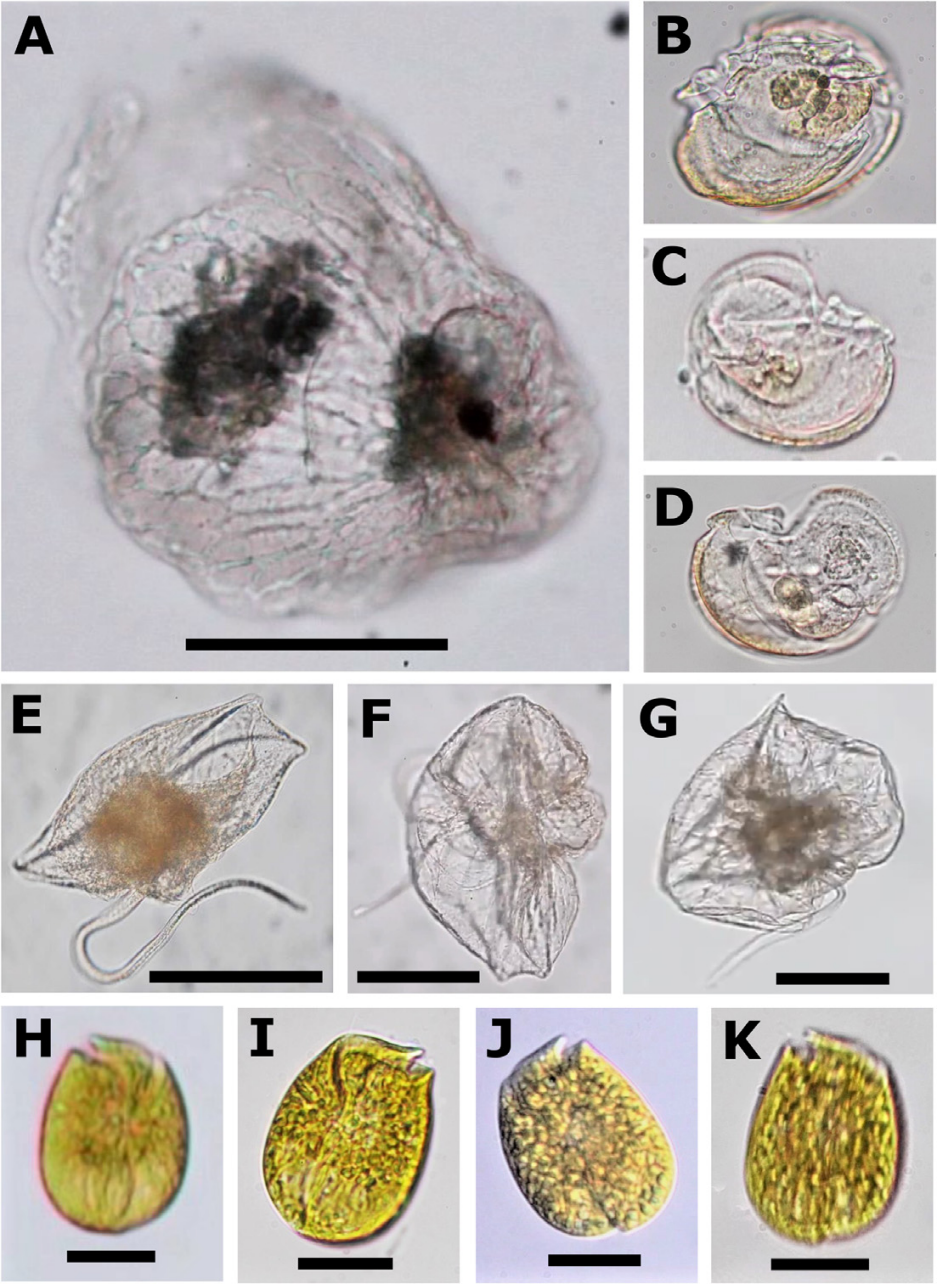
Light micrographs of cells collected for single cell transcriptomics. (A) Bell-shaped Abediniales cell Ab–JP. (B and C) *F. amicum* gen. nov. cells Fa-JP1, Fa-JP2, and Fa-FC3, respectively. All three images are to scale with panel A. (E to G) *Noctiluca* cells No-FC1, No-FC2, and No-FC3. (H to K) *Amphidnium* sp. cells Am-JP1, Am-JP2, Am-JP3, and Am-JP4. Scale bars in are 100 µm in A to G and 25 µm in H to K. See supplementary spreadsheet and videos for more detailed information pertaining to each cell.

While *Noctiluca, Spatulodinium, Kofoidinium*, and *Amphidinium* cells were immediately recognizable based on morphology (35, 28, 38, 39), two morphotypes we observed could not be assigned to any described taxon. The shape of the first morphotype, labeled Ab–JP, was difficult to discern as the cell was unmoving and possibly contorted (Fig. 1A; Supplementary video 1—this and all subsequent videos are available at https://doi.org/10.5281/zenodo.6326522). Mostly hyaline throughout, the cell appeared to be flattened with a thick center, similar to the body plan of *Abedinium* (36). However, the peripheral shape of the cell could not be determined, as it was contracted into a bell shape. This medusa-like form has been described in *Pomatodinium, Leptodiscus*, and *Cymbodinium* (33, 34, 40), but no molecular data from these taxa are available for comparison. The edges of the cell resembled the undulated contractile margins of *Abedinium*, and a spot of bright red pigmentation was also visible in the thick center of the cell near a cluster of food vacuoles (31). Unlike in *Abedinium*, a network of refringent, contiguous channels radiated from the center of Ab–JP to its margins. No tentacles, flagella, or trichocysts were visible.

**Fig. 2.**
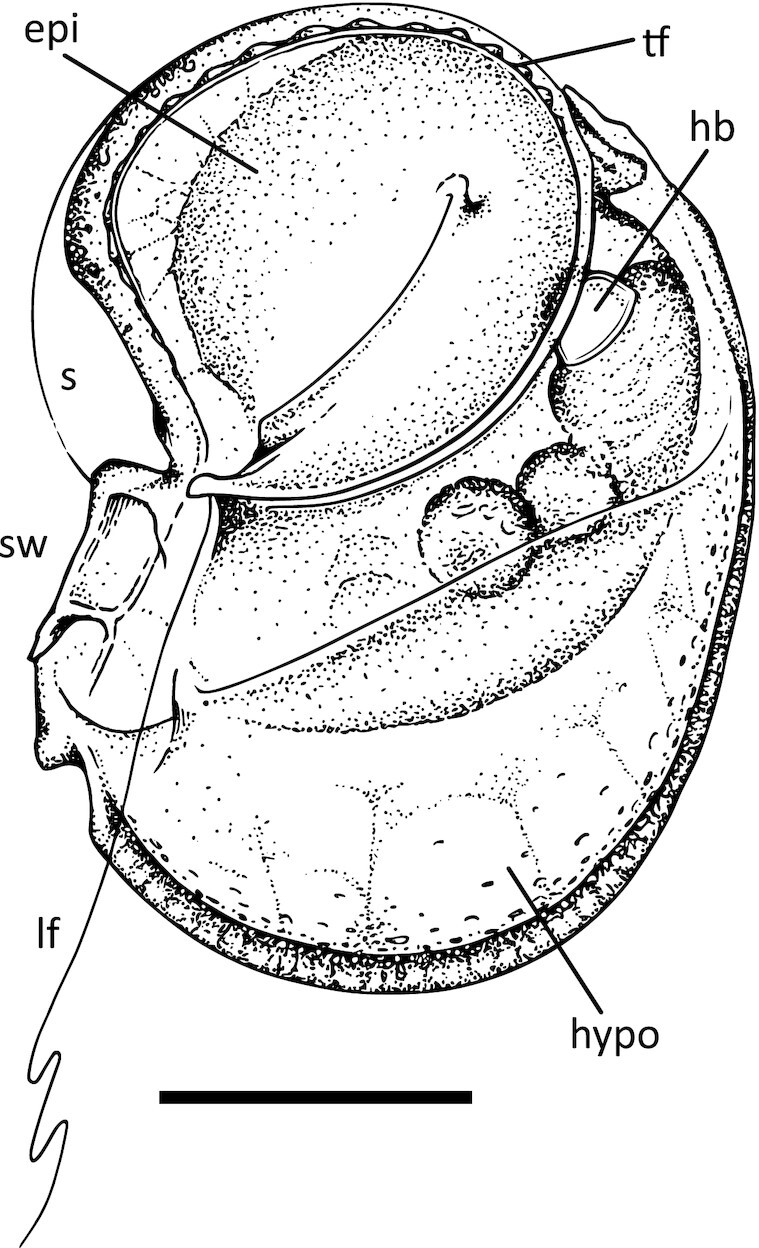
Schematic illustration of *F. amicum* gen. nov. sp. nov. cell based on combined observations of cells Fa-JP1, JP2, and FC3. Structures indicated are: epi = episome; tf = transverse flagellum; hb = hyaline body; s = shell; sw = sulcal wing; lf = longitudinal flagellum; and hypo = hyposome. Scale bar is 25 µm.

The second morphotype was observed in three different isolated cells, labeled Fa-JP1, Fa-JP2, and Fa-FC3 (Fig. 1B to D). These share an overall similarity to morphological descriptions of *Kofoidinium* sporonts (35), but with some pronounced differences. Like *K. pavillardii*, cells Fa-JP2 and Fa-FC3 were laterally flattened and asymmetrical, usually laying prone on the sample vessel floor, but occasionally swimming upright (Fig. 1C and D; Supplementary video 2). The shape of cell Fa-JP1 is more ambiguous, seemingly bent or medially swollen (Fig. 1B). Cells are ∼50 µm wide and 95 µm long. In all specimens, the edge of what appears to be a transparent shell-like structure similar to that of *Kofoidinium* was visible as a delicate line departing from the curved anterior, arcing back toward the cell and intersecting with the mid-point of its ventral edge (Fig. 2). It is not clear if the perimeter of this shell continues in a full circle to form a shield-like structure like it does in *Kofoidinium*. As in *K. pavillardii*, the ruffle-like transverse flagellum is anchored ventrally, rounding the anterior of the cell and curving back across the cell body. This flagellum lies within a cingulum that delineates the horseshoe-shaped boundary of a flat and laterally-facing episome, creased from the sulcus to the halfway point between the center and the dorsal edge. The episomes of cells Fa-JP2 and Fa-FC3 faced opposite sides, showing that this asymmetry can occur in either left- or right-facing orientations. The presence of a prominent sulcal wing is notably different from *Kofoidinium* sporonts. A longitudinal flagellum runs adjacent to this structure down the sulcal groove toward the posterior, extending behind the cell while swimming. Inside all three cells, a reflective, hyaline, and orb-like organelle was positioned dorso-anteriorly, adjacent to a cluster of food vacuoles (Fig. 2). Diffuse, brown coloration is visible at the anterior edge of the cell; however, this is likely a lighting artifact, as the locality of this color shifts as the cell rotates (Supplementary video 2). When still, cells lay prone, reeling in the flagellum and casting it back out in an unfurling motion accompanied by a current that propels particles down its length (Supplementary video 2). While more study is needed to determine if this taxon is either planktonic or benthic, these cells were only observed in samples that contained some benthic sediment and other known benthic taxa. This, together with observations of stationary “fishing” behavior and the inclination of all cells to remain close to the substrate, presents the intriguing possibility that *Fabadinium* gen. nov. is the first described benthic noctilucoid. Based on these characteristics (and molecular phylogeny described below), we conclude that these cells represent a new genus and species, *F. amicum* (see taxonomic summary). Over months of searching, only three *F. amicum* cells were found and destroyed to generate sequencing data, leaving no physical specimens available for preservation.

**Fig. 3.**
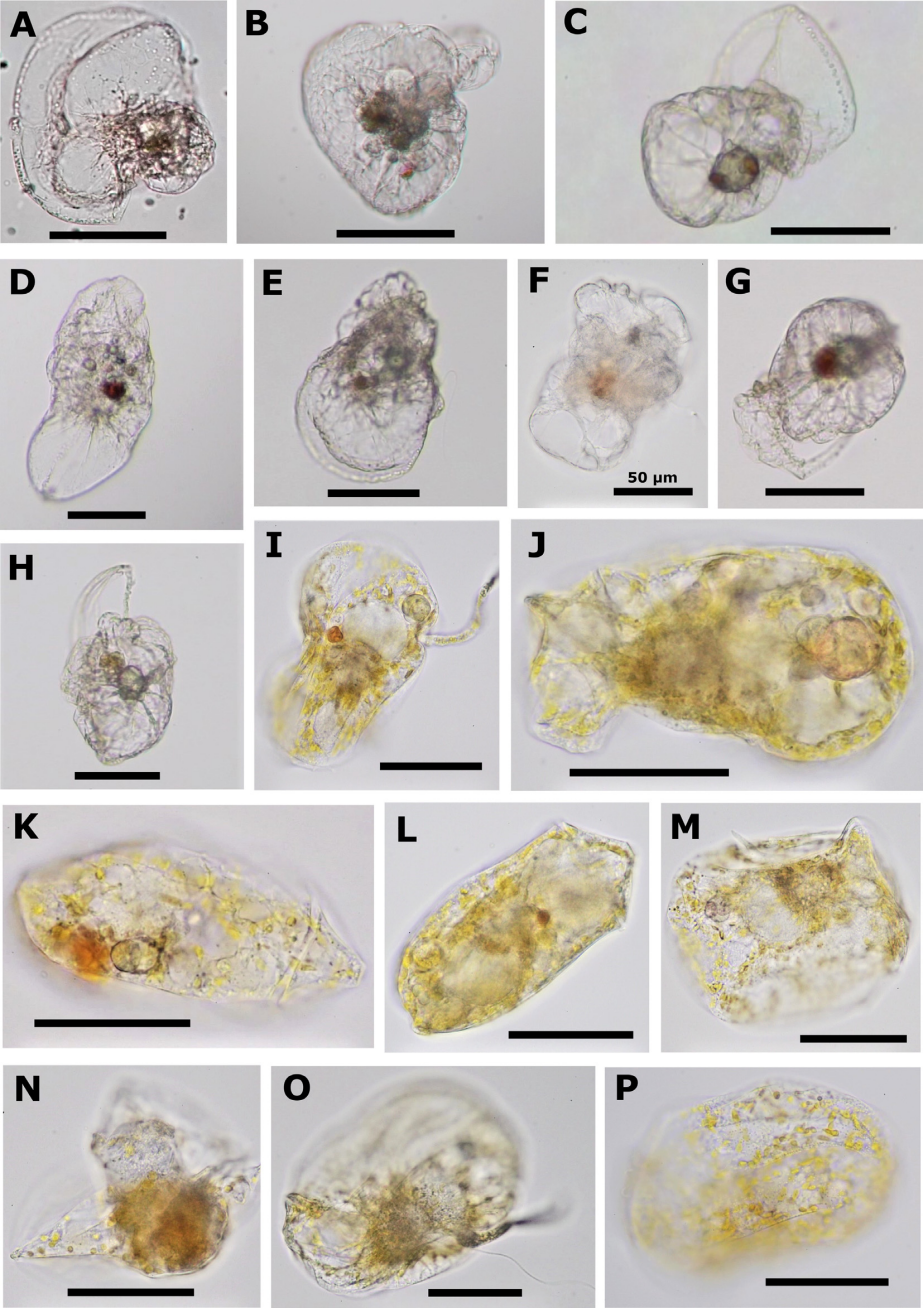
Light micrographs of cells collected for single cell transcriptomics. (A to H) *Kofoidinium* cells Ko-JP1, Ko-FC2, and Ko-QI3-8. (I to P) *S. pseudonoctiluca* cells Sp-QI1 to 8. All scale bars are 100 µm unless otherwise labeled. See supplementary spreadsheet and videos for more detailed information pertaining to each cell.

*Noctiluca, Kofoidnium*, and *Spatulodinium* cells assumed a range of morphologies upon collection, but all had clear morphological characteristics consistent with previously described species of these genera. The three *Noctiluca* cells appeared to be in various stages of expansion to form the typical globular vegetative morphology (Fig. 1E to G). In *Kofoidinium*, individuals changed shape during observation due to the dynamic velum (a modified hyposome) characteristic of this genus [(41); Fig. 3A to H; Supplementary video 3]. A thread-like, trailing flagellum terminated by a thick tip was observed in Ko-JP1, growing longer as the cell drifted. In Ko-FC2 and Ko-QI3, a ruffle-like flagellum resembling the typical dinoflagellate transverse flagellum could be seen hugging the outer surface of the cell. In two cases (Ko-QI3; QI5), long fibers appearing thicker than the thin flagellum in JP1 were observed near the cell, although whether they had once been attached could not be confirmed. Only Ko-QI5 possessed a flagellum resembling and beating like a typical dinoflagellate longitudinal flagellum. All individuals carried at least one small reddish to orange pigmented body in the part of the cell where food vacuoles were concentrated. Two individuals (Ko-QI7; QI8) shared a distinct ring-like morphology that differed from the rest of the cells, possibly representing an undescribed life stage (Fig. 3G and H). No cells were observed to have the circular shell-like structure observed in some *Kofoidinium* specimens (35).

The diverse morphologies of *Spatulodinium* reflect a range of life stages (Fig. 3I to P; Supplementary video 4). Three individuals (Sp-QI1; QI5; and QI7; Fig. 3I, M, and O) possessed a tentacle, with Sp-QI5 seemingly in the process of forming the appendage. These cells were likely at or near maturity, while the rest were in or transitioning out of the immature gymnodinoid stage (35, 38). All cells contained plastids dispersed throughout the cell, including the tentacle when present. Longitudinal and ruffle-like transverse flagella were visible in all cells except Sp-QI5 (only longitudinal) and Sp-QI6. In Sp-QI2, the putative transverse flagellum was reduced and slow-beating. Some individuals possessed a pigmented body nearly identical in size, shape, and color to those seen in *Kofoidinium*. In contrast to *Spatulodinium* and *Kofoidinium, Amphidinium* sp. cells were all uniformly squarish ovoid in shape and dorso-ventrally flat with yellowish-green plastids (Supplementary video 5).

### Phylogenomic analysis of noctilucales

**Fig. 4.**
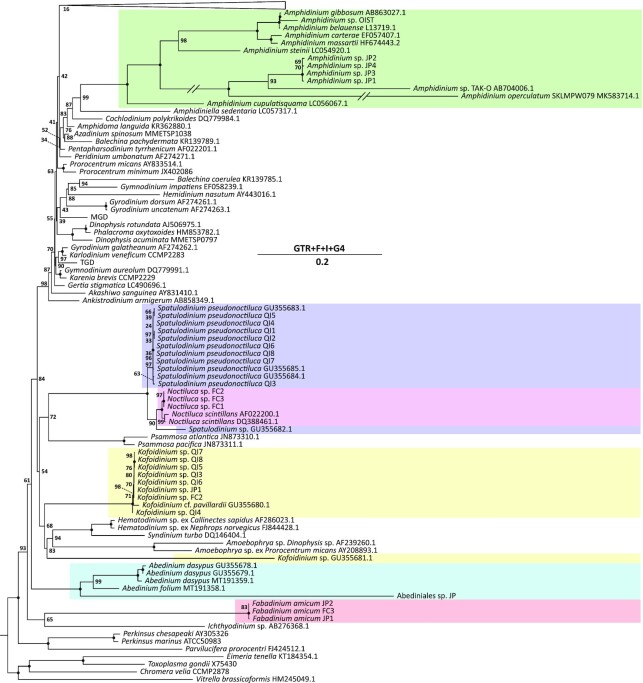
Maximum likelihood phylogeny inferred from dinoflagellate and close relative SSU rRNA gene sequences. Node values represent bootstrap support with black circles indicating 100. Colored boxes highlight existing data for each relevant taxonomic group and correspond to the groups highlighted in Fig. 5. Shortened branches are half the original length. Scale bar represents the estimated nucleic acid substitutions per site and includes the substitution model. Collapsed branches contain only core dinoflagellate taxa—full tree available at https://doi.org/10.5281/zenodo.6326522.

*Kofoidinium, Noctiluca, Amphidinium*, and *S. pseudonoctiluca* grouped closely with other members of their genera in a phylogenetic tree inferred from SSU rDNA sequences. Cell Ab–JP branched with the Abediniales with full support, but was not highly similar to any existing sample, consistent with its distinctive morphological characteristics. The three *Fabadinium* cells branched together with strong support, but their position in the tree was unresolved (Fig. 4). In searches against Genbank, Tara Oceans, and benthic environmental databases, SSU rDNA sequences from *Fabadinium* returned no highly similar hits (≥99% identity), consistent with this being a previously undescribed and rare taxon.

**Fig. 5.**
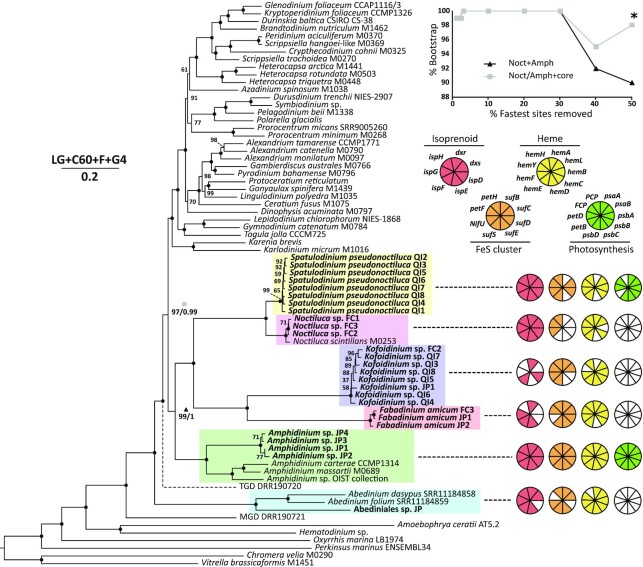
Maximum likelihood phylogeny of dinoflagellates based on 205 genes (each gene present in ≥60% of taxa) translated to amino acid sequences, showing the presence of plastid biosynthesis and photosynthesis-related genes in Noctilucales, *Amphidinium*, and Abediniales lineages. Taxa introduced in the present study are shown in bold. Colored boxes highlight relevant taxonomic groups, corresponding with Fig. 4. Node values represent bootstrap support with black circles indicating 100. Two nodes also include a second value representing Bayesian posterior probability. The branch for TGD is dotted because it branched in a different position in Bayesian analyses (between MGD and the Abediniales lineage) with full support. Taxa with alphanumeric identifiers beginning with “M” are from the Marine Microbial Eukaryotic Transcriptome Sequencing Project (48). Scale bar indicates the number of amino acid substitutions per site and is accompanied by the substitution model used to infer the phylogeny. Line graph shows the change in bootstrap support of two nodes (indicated with marker shapes) as a function of fast-evolving site removal. The asterisk is present because one branch from the core dinoflagellates was displaced in this topology (see Supplementary Fig. S1). Pie charts show the presence of plastidial isoprenoid (red), iron-sulfur (FeS) cluster (orange), and heme (yellow) biosynthesis pathway enzymes and photosynthesis related genes (green) from the combined data in each lineage.

Each single cell transcriptome was included in a multigene phylogenomic analysis to elucidate the relationships in and around the Noctilucales lineage. Assembly quality varied, but at least one transcriptome from each taxonomic group containing over 50% of conserved alveolate genes in BUSCO v5 coverage estimates, with the exception of Abediniales [Supplementary spreadsheet; (42)]. In a 205 gene ML phylogeny, all relationships within the Noctilucales were resolved with full statistical support (Fig. 5). *Noctiluca* and *S. pseudonoctiluca* branched closely in a lineage sister to *Kofoidinium*, confirming SSU analyses that concluded *Spatulodinium* belongs to Noctilucaceae instead of Kofoidniaceae (30). *Fabadinium* gen. nov. and *Kofoidinium* were both long branches in the same lineage, suggesting that the morphological similarities between these groups noted above are homologous.

More importantly, we found the position of the Noctilucales within the dinoflagellates as a whole to be in conflict with previous analyses inferred from single genes or including only a single taxon representing the Noctilucales. Specifically, rather than forming a deep branch that is sister to other core dinoflagellates, phylogenomic trees all showed Noctilucales to form a monophyletic group with *Amphidinium*. Bootstrap support for this relationship was strong, at 99%. However, this value increased to full support after removing the top 2% of fast evolving sites in the alignment (Fig. 5). Bayesian analysis and all topology tests also confirmed this relationship, although the placement of the enigmatic “TGD” dinoflagellate was different in ML and Bayesian analyses (Supplementary Fig. S1).

The contrasting morphologies and life histories between *Amphidinium* and the Noctilucales have traditionally led taxonomists to regard these lineages as not particularly closely related. Members of the *Amphidinium* genus tend to be dorso-ventrally flat, inhabiting benthic and interstitial habitats (43), while the Noctilucales are planktonic, relatively large, and very diverse in shape. Phylogenetic analyses using rRNA and other conserved gene sequences have generally supported the idea they are not closely related, placing Noctilucales as more deep-branching, but rarely with strong support (44). Due to the instability of *Noctiluca scintillans* in SSU phylogenies, Saldarriaga et al. (45) questioned the deep-branching position of the Noctilucales, noting that *Noctiluca* shares some rare traits with the core dinoflagellate genus *Gymnodinium*, while the sporonts of *K. splendens, K. velelloides*, and *Spatulodinium* bear a strong resemblance to many athecate taxa that possess an anterior transverse girdle and ventral sulcus (35, 41). The flattened morphology of *Fabadinium* gen. nov. is also more like the benthic *Amphidinium* sensu stricto body plan than those of its more globular relatives. In our study, the inclusion of more representative taxa and orthologous genes is likely responsible for the emergence of an *Amphidinium* and Noctilucales sisterhood, because this relationship did not materialize in earlier studies. Outstanding questions still remain; however, like determining whether other members of Leptodiscaceae are members of the Noctilucales, or perhaps related to Abediniales. Broader transcriptomic sampling of the extensive and diverse *Amphidinium* clade will also be crucial for verifying the sisterhood of this lineage to the Noctilucales (46, 47). Similarly, the addition of *Gyrodinium* will be of interest, as earlier analyses indicate a relationship between this group and *Amphidinium* (49, 45).

### Noctilucales have ancestral plastids

Until recently, the Noctilucales were thought to be an exclusively heterotrophic group lacking plastids. However, with the advent of transcriptome sequencing, plastidial genes for known plastid biosynthetic pathways for heme, isoprenoid, and iron–sulfur cluster synthesis have been found in a variety of heterotrophic dinoflagellates, including *Noctiluca* (50, 23). Although photosynthesis and the plastid genome have been lost in heterotrophic dinoflagellate lineages, these genes were long ago moved to the host nucleus, and the transcripts retain a distinctive N-terminal extension that targets the protein product back to the organelle (51, 52). Transcriptomes from every lineage presented here included transcripts from genes involved in these pathways (Fig. 5). Not all transcripts appeared in every individual transcriptome, likely due to low expression and incomplete sampling. Phylogenies for each gene show that all transcripts group with known dinoflagellate sequences of peridinin plastid origin (https://doi.org/10.5061/dryad.ngf1vhhw7).

Historically, noctilucoids have been presumed to lack plastids, and in this context the yellow-green coloration of *Spatulodinium* was first attributed to lipid storage instead of photosynthetic pigments (35). However, observations of chlorophyll epifluorescence in recent years have confirmed that chloroplasts are present in this taxon (30). But because dinoflagellates have a propensity for adopting new plastids (19), and some noctilucoids have been shown to harbor photosynthetic symbionts (53), the exact nature of *Spatulodinium* plastids have remained uncertain. Our phylogenetic analyses of plastid-related transcripts now show that *S. pseudonoctiluca* plastids are of the ancestral peridinin type (Supplementary Fig. S2). To verify that these plastids are photosynthetic, we searched for a selection of protein coding genes involved in photosystems I and II, the cytochrome *b6f* complex, and the chlorophyll *a/c* binding proteins, fucoxanthin- and peridinin-chlorophyll protein (FCP and PCP, respectively). All but two of these genes, *petB* and PCP, were recovered from *Spatulodinium* transcriptomes. *Spatulodinium* sequences consistently clustered with homologues from photosynthetic, peridinin-containing dinoflagellates (Supplementary Fig. S2). The absence of PCP raises the interesting question of whether *Spatulodinium* produces the carotenoid, peridinin.

The possibility that plastid sequences were the result of contamination from consumed prey or the ambient environment was refuted by the complete absence of photosynthetic transcripts in any of the nonphotosynthetic noctilucoid transcriptomes. While contaminant sequences cannot be completely eliminated in most single cell transcriptomes, they are typically sparse and fragmented compared to host sequences and can be easily distinguished from the host in phylogenetic trees [e.g. see (54)].

### Unique noctilucoid traits are derived, rather than ancestral

Previous phylogenetic analyses mostly showed Noctilucales to be a deep-branching group sister to the core dinoflagellates, and based on their unique traits, were thought to be ancestral. Current analyses now call this into question, because of the increase in both the number of positions available and the taxonomic breadth of Noctilucales and other lineages in the analyses. The inclusion here of several new genera of Noctilucales contributes to this, as do two recently described deep-branching species, TGD and MGD (55), and Abediniales (37), all of which now branch more deeply than Noctilucales. Although a comprehensive study on the life cycle of Abediniales, MGD, and TGD have yet to be conducted, transmission electron microscopy has shown that MGD at least has a condensed chromosome nucleus in its vegetative life stage (55). In all topologies MGD branches sister to the group containing Noctilucales, and this alone suggests that permanently condensed chromosomes have been lost in the Noctilucales.

However, the potential sisterhood between Noctilucales and *Amphidinium* provides even more compelling evidence that this and all other unique morphological characteristics in Noctilucales are derived rather than ancestral. Several members of the genus *Amphidinium* are well-studied and possess many traits common in core dinoflagellates. This mostly photosynthetic genus seemingly contrasts with the mostly heterotrophic Noctilucales, but some *Amphidinium* sensu stricto do lack photosynthesis (56, 57). In addition to having condensed chromosomes in the vegetative life stage (58, 23, 59), *Amphidinium* species also have the ruffle-like transverse flagellum attached along its length to the cell, which is notably absent from all *Noctiluca* life stages (60, 61). While it has been proposed this feature evolved after the branching of Noctilucales (23), this was based on a phylogenomic analysis in which *Noctiluca* was the sole representative of its lineage. In fact, the ruffle-like transverse flagellum has been observed in other noctilucoids, including species confirmed in the present study to be members of the Noctilucales [Supplementary videos 2 and 3; (35)]. Thus, this feature was likely lost even more recently, in the ancestor of *Noctiluca*.

## Conclusion

Even though the vastly different life strategies and body plans of Noctilucales and *Amphidinium* seem at odds, molecular analysis suggests these groups are members of the same lineage. Noctilucales and *Amphidinium* also occupy very different realms of the aquatic environment, but benthic and planktonic associations have arisen independently in other dinoflagellate groups (62, 63), and morphological similarities have been observed between noctilucoid life stages and *Amphidinium* (35, 46). While more transcriptomes of related taxa will inevitably continue adding resolution to these relationships, the present study highlights the importance of single cell transcriptome sequencing for discovering unexpected relationships and learning about character evolution in rare and uncultured taxa.

### Taxonomic summary

Class Dinophyceae West and Fritch, 1927; order Noctilucales Haeckel, 1894; family Kofoidiniaceae (Cachon and Cachon) Taylor, 1976; and genus Fabadinium gen. nov. Cooney, Leander, and Keeling, 2022.

#### Diagnosis

Forms a long branch sister to *Kofoidinium* with full support. Cells lack pigment, are athecate, and in at least one stage of the life cycle, have a laterally flattened body plan with the episome tilted to face one side or the other. In this form, the morphology of the cell is static.

#### Type species

*Fabadinium amicum* Cooney, Leander, and Keeling 2021

#### Etymology

The Latin “faba” and “dinium” translate to bean and vortex, respectively. The prefix refers to the shape of the cell, which resembles a lima bean.

#### Zoobank ID

5DC0A65F-4DB2-4B30-A9DC-44885EF36501

*Fabadinium amicum* sp. nov. Cooney, Leander, and Keeling, 2022

#### Diagnosis

Unicellular heterotroph with a static, asymmetric, laterally flattened body plan in at least one stage of life. Possesses a ruffle-like transverse flagellum attached along its length to the cell, and a trailing longitudinal flagellum. Cells are approximately 50 µm wide and 95 µm long and have a prominent wing-shaped projection adjacent to the sulcus.

#### Holotype

Cell Fa-JP2, seen in Fig. 1C (accession OP161899).

#### Etymology

The Latin “amicum” means friend.

#### Type locality

Obtained from Jericho Pier in Vancouver, BC (49.276996, −123.201612). Collector: E. Cooney.

#### Sequence data

Raw reads: SRA (project number PRJNA859917). Single-cell transcriptome: https://doi.org/10.5061/dryad.ngf1vhhw7. SSU rRNA gene sequence: GenBank accession number OP161899.

#### Zoobank ID

D03A6A2F-5FE2-429C-8EFE-2F444B076508

## Materials and methods

### Single cell collection and imaging

Sampling took place from the shore of English Bay in Vancouver, and on the northwest side of Quadra Island in British Columbia. In Vancouver, net tows (20 µm mesh) were deployed from two sites: a public boat dock at Snauq (also known as False Creek; 49.277010, −123.139921), and Jericho Beach Pier (49.276996, −123.201612). Sampling was conducted on an approximately weekly basis from May 2020 to September 2021 in an ongoing search for rare and understudied dinoflagellate taxa. Sampling on Quadra Island took place in August and September of 2021. Sampling dates and cell IDs can be found in the supplementary spreadsheet (Supplementary Material online).

During sampling on December 10, January 8, and January 22, the net was lowered to the sea floor below the dock and allowed to pick up small amounts of sediment before towing through the water column. Within an hour after every collection, water samples were observed using a Leica DM IL microscope. Cells were isolated using a microcapillary pipet and washed in sea water filtered from the source sample using a 0.2 µm syringe filter. During this process, cells were imaged (stills and video) using a Sony A7r III before being placed into lysis buffer (64) and stored at −70°C.

### Sequencing and transcriptome assembly

Isolated cells were processed to make Illumina cDNA libraries according to the Smart-seq2 protocol (54, 64). Libraries were sequenced on the Nextseq platform at the Sequencing and Bioinfomatics Consortium, University of British, Columbia. The resulting paired-end reads were trimmed using Cutadapt (65) and then assembled with rnaSPAdes v3.15.1 (66). Removal of bacterial, metazoan, and diatom contamination was performed on assemblies by using Blastx and Blastn (67) to search NCBI nt and Uniprot reference proteomes, respectively (The Uniprot Consortium 2021). Contaminant contigs were then identified using BlobTools and removed (68). Open reading frame identification and annotation were performed using TransDecoder v5.5.0 (69) and BlastP with an e-value threshold of ≤1e10^−5^ to search against the Uniprot database (70).

### Identification, environmental surveys, and phylogenomic analysis

Taxonomic classifications were determined with a combination of literature search and SSU rDNA sequence comparison. The most complete SSU sequences were extracted from each transcriptome and used as queries to perform megaBLAST searches against Genbank. They were then aligned with other dinoflagellate and close relative SSUs using MAFFT v.7.481 (71). This alignment was trimmed at a 30% gap threshold with trimAl v.3 (72) before generating a tree in IQ-TREE v1.6.12, using ModelFinder to select the best fit model GTR + F + I + G4 (73, 74). Searches using SSU sequence queries were also performed against the Marine Atlas of Tara Oceans Unigenes (75, 76) and a curated database of 113,203,549 benthic environmental sequences (see Supplementary Material for list of benthic data sources).

To perform a multigene phylogenomic analysis, 263 curated gene alignments (77) were queried against each transcriptome in a series of BlastP searches. The resulting hits were aligned with their respective queries using MAFFT-LINSI. Each alignment was trimmed with an 80% gap threshold in trimAl before single gene tree construction with IQ-TREE (model: LG + G). Contaminants, paralogs, and isoforms were removed from each alignment after visual inspection of its respective tree. Cleaned alignments, containing at most one representative sequence from each transcriptome, were parsed using SCaFoS v4.55 (78), keeping only relevant taxa and the genes present in ≥60% of them. A maximum likelihood (ML) phylogeny was inferred from a final concatenated alignment of 205 genes in IQ-TREE, using the model LG + C60 + F + G4. A Bayesian analysis was also performed on this alignment in four parallel runs in Phylobayes-MPI, using the model CAT + GTR + G4 [(79), Phylobayes manual v4.1]. Chains were run until they had passed 10,000 iterations and yielded a maxdiff <0.3. The first 20% of trees were removed as burn-in and a consensus tree was constructed from every second tree of each parallel run. Alternative topologies to that of the ML analysis were ruled out in a series of topology tests (Supplementary Table S1). To determine the effect of fast-evolving sites on node support, the fastest evolving sites were incrementally removed up to 50%.

### Plastid protein characterization

Using BlastP, all transcriptomes were searched for homologs of plastidial heme, isoprenoid, and iron–sulfur cluster biosynthesis pathway enzymes, and a collection of photosynthesis-related genes. Known dinoflagellate homologs were used as queries and an e-value threshold of ≤1e^−25^ was applied for each search. Blast hits for pathway enzymes were added to pre-existing curated alignments (50) from which ML trees were inferred to verify their identity and eliminate contaminant sequences. Blast hits for photosynthesis genes were added to alignments curated in this study, with the exception of FCP, which was searched against Genbank using BlastP to verify dinoflagellate plastid origin.

## Supplementary Material

pgac202_Supplemental_Files

## Data Availability

The data underlying this article are available in Genbank (accession numbers OP161893-OP161919), SRA (project number PRJNA859917), in the Supplementary Material online, and at https://doi.org/10.5061/dryad.ngf1vhhw7 and https://doi.org/10.5281/zenodo.6326522.
