# Supplementary material for: Phylogenomics shows unique traits in Noctilucales are derived rather than ancestral
Source: PNAS Nexus. 2022 Sep 22;1(4):pgac202. doi: 10.1093/pnasnexus/pgac202 (PMC9802342; doi:10.1093/pnasnexus/pgac202)
Supplement: pgac202_Supplemental_Files [file pgac202_supplemental_files.zip › PNASNEXUS-PNASNEXUS-2022-00515-s01.docx]

**
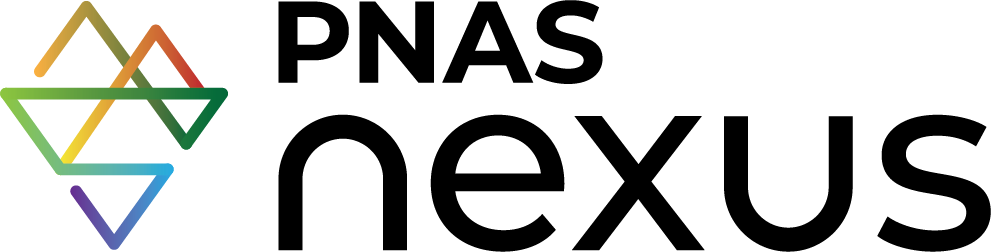
**

**Supplementary Information for**

Phylogenomics shows unique traits in Noctilucales are derived rather than ancestral.

Elizabeth C. Cooney, Brian S. Leander, Patrick J. Keeling

Corresponding author: Elizabeth C. Cooney

Email: [lizcooney](mailto:xxxxx@xxxx.xxx)22@gmail.com

**This PDF file includes:**

Table S1

Figures S1 to S2

Video captions

SI References

Benthic database sources

**Other supplementary materials for this manuscript include the following:**

At [https://doi.org/10.5281/zenodo.6326522](https://doi.org/10.5281/zenodo.6326522):

Videos S1 to S5

Full SSU tree

At <https://doi.org/10.5061/dryad.ngf1vhhw7>:

Supplementary spreadsheet

Peptide transcriptome assemblies

Plastid gene trees

Multiprotein alignment

**Table S1.** Tree topology test p-value outputs. Topology (“topo”) 1 matches the maximum likelihood (ML) tree reported in the main text. Alternative topologies in this table are shown below. deltaL = logL difference from the maximal logl in the set. bp-RELL = bootstrap proportion using RELL method (Kishino et al. 1990). p-KH = p-value of one sided Kishino-Hasegawa test (Kishino & Hasegawa 1989). p-SH = p-value of Shimodaira-Hasegawa test (Shimodaira & Hasegawa 1999). c-ELW = Expected Likelihood Weight (Strimmer & Rambaut 2001). p-AU = p-value of approximately unbiased (AU) test (Shimodaira 2002). Plus signs denote the 95% confidence sets. Minus signs denote significant exclusion. All tests performed 10000 resamplings using the RELL method.

| Topo | logL | deltaL | bp-RELL |  | p-KH |  | p-SH |  | p-WKH |  | p-WSH |  | c-ELW |  | p-AU |  |
| --- | --- | --- | --- | --- | --- | --- | --- | --- | --- | --- | --- | --- | --- | --- | --- | --- |
| 1 | -1507834.918 | 0 | 0.994 | + | 0.992 | + | 1 | + | 0.992 | + | 1 | + | 0.994 | + | 0.996 | + |
| 2 | -1508018.156 | 183.24 | 0 | - | 0 | - | 0.191 | + | 0 | - | 0 | - | 3E-49 | - | 5.06E-09 | - |
| 3 | -1508424.883 | 589.96 | 0 | - | 0 | - | 0.0008 | - | 0 | - | 0 | - | 8.72E-189 | - | 6.87E-41 | - |
| 4 | -1508342.249 | 507.33 | 0 | - | 0 | - | 0.0035 | - | 0 | - | 0 | - | 3.15E-147 | - | 4.62E-48 | - |
| 5 | -1507971.396 | 136.48 | 0 | - | 0 | - | 0.262 | + | 0 | - | 0 | - | 1.15E-19 | - | 4.31E-57 | - |
| 6 | -1519658.511 | 11824 | 0 | - | 0 | - | 0 | - | 0 | - | 0 | - | 0 | - | 6.16E-10 | - |
| 7 | -1519633.976 | 11799 | 0 | - | 0 | - | 0 | - | 0 | - | 0 | - | 0 | - | 2.54E-09 | - |
| 8 | -1519654.536 | 11820 | 0 | - | 0 | - | 0 | - | 0 | - | 0 | - | 0 | - | 1.04E-10 | - |
| 9 | -1507895.153 | 60.235 | 0.0061 | - | 0.0082 | - | 0.474 | + | 0.0082 | - | 0.0265 | - | 0.00603 | - | 0.00452 | - |

**Alternative topologies corresponding with Table S1**:


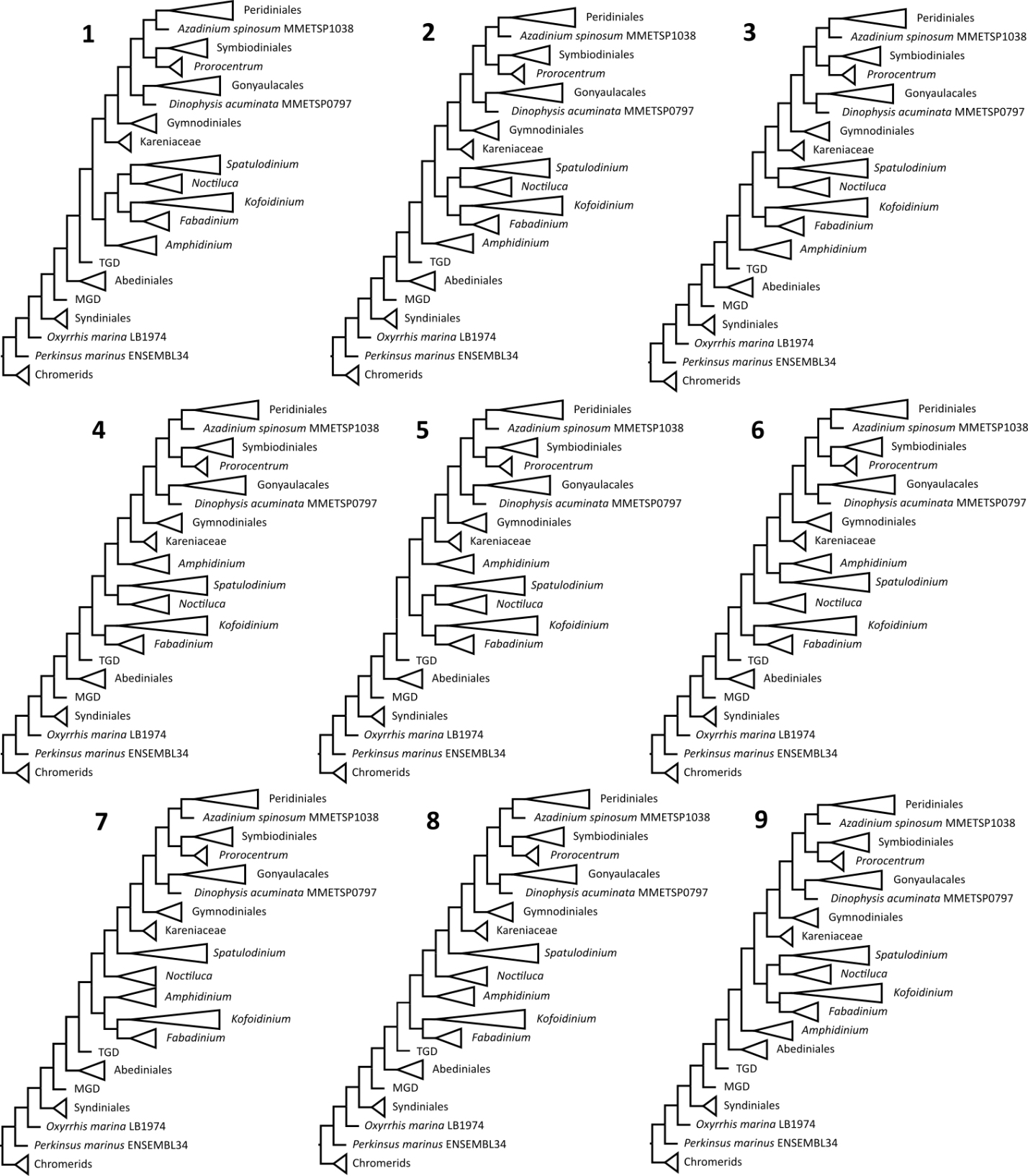


**
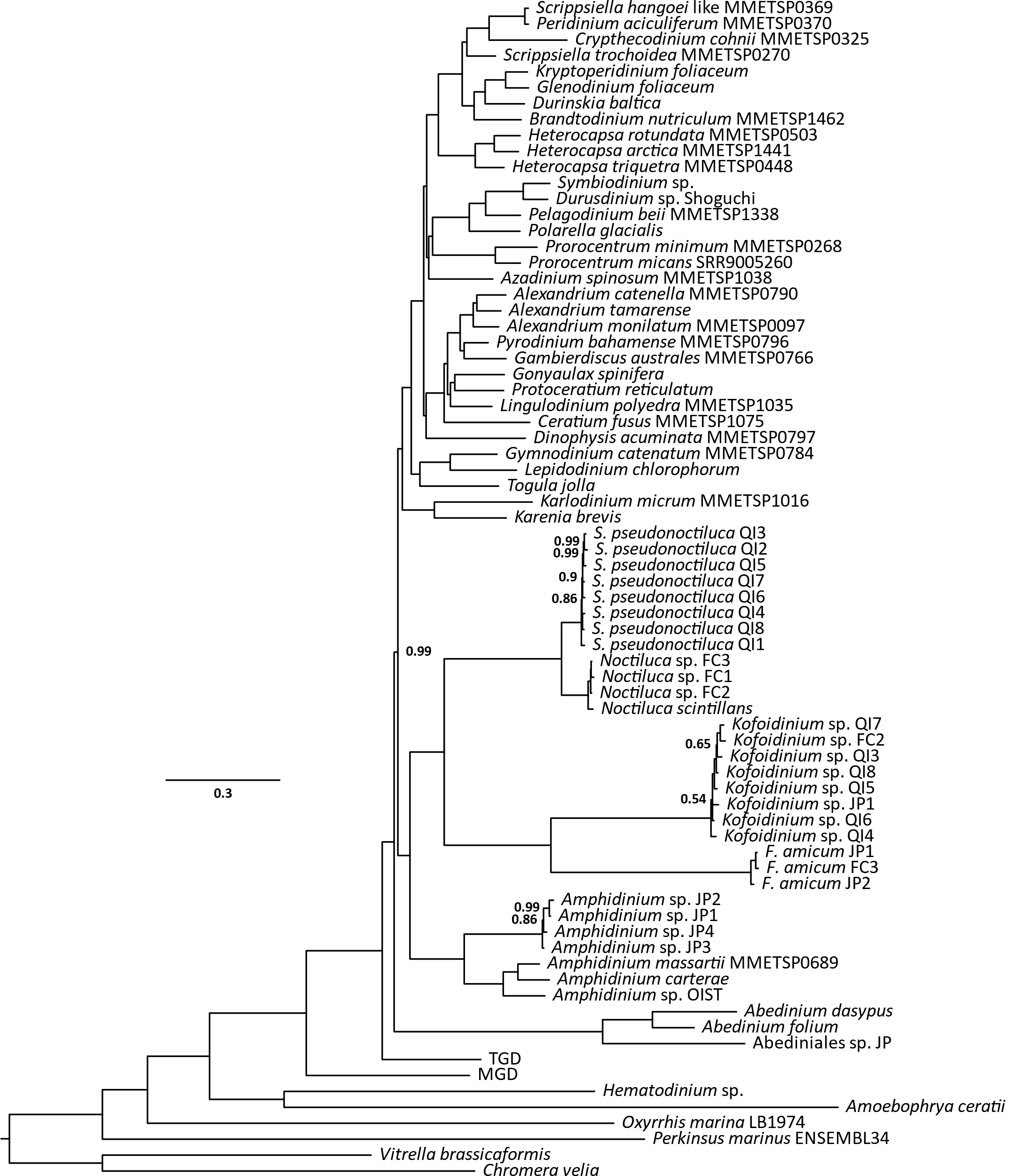
**

**Fig. S1.** Consensus tree of a Bayesian analysis run in four parallel chains from the same alignment as the maximum likelihood (ML) analysis in the main text. Note the placement of TGD, which is different than in the ML phylogeny. Node values represent posterior probability support. Nodes with no values are fully supported. Scale bar represents amino acid substitution frequency.

**Fig. S2.** Maximum likelihood phylogenies of six photosynthetic genes (petD, psaA, psaB, psbA, psbB, psbC) found in *Spatulodinium pseudonoctiluca*. All shortened branches are half their original length. Pink = *S. pseudonoctiluca*; cyan = *Amphidinium* spp.; green = the rest of the dinoflagellates.

**
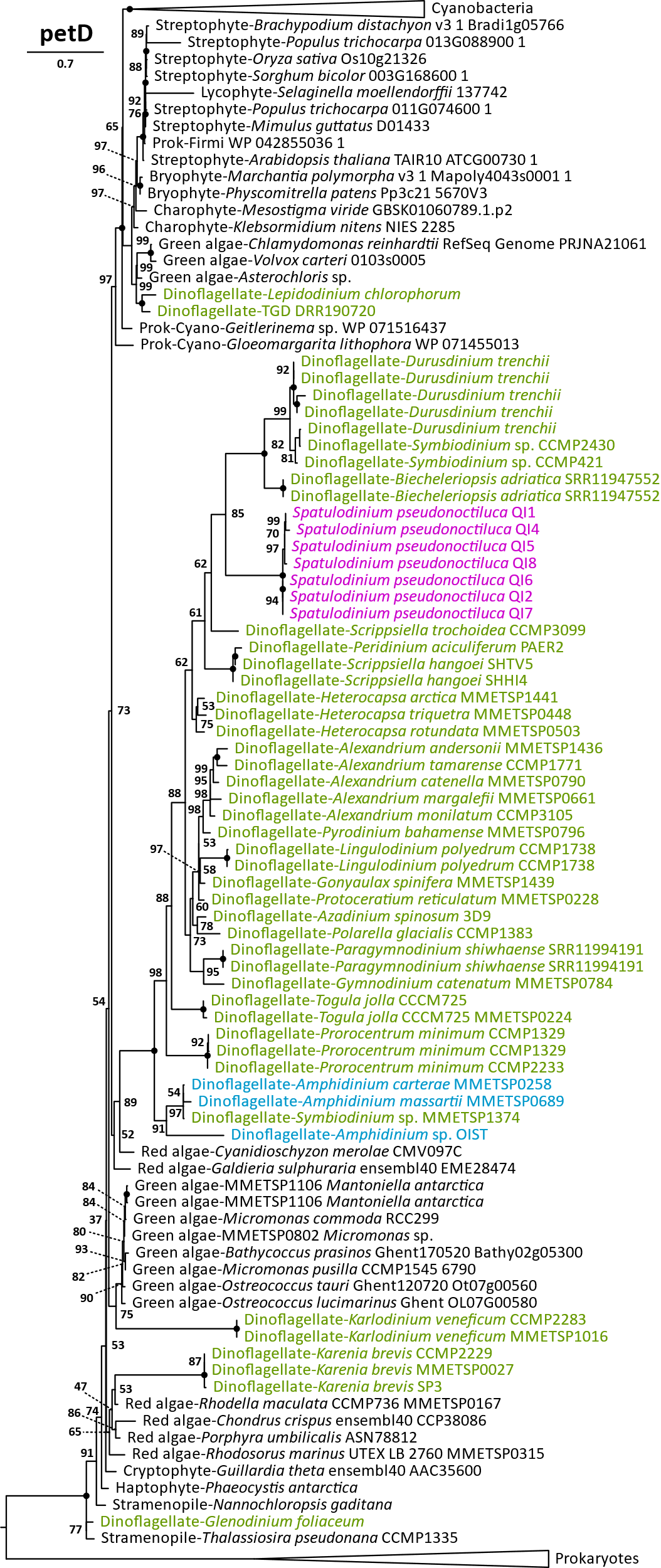
**

**
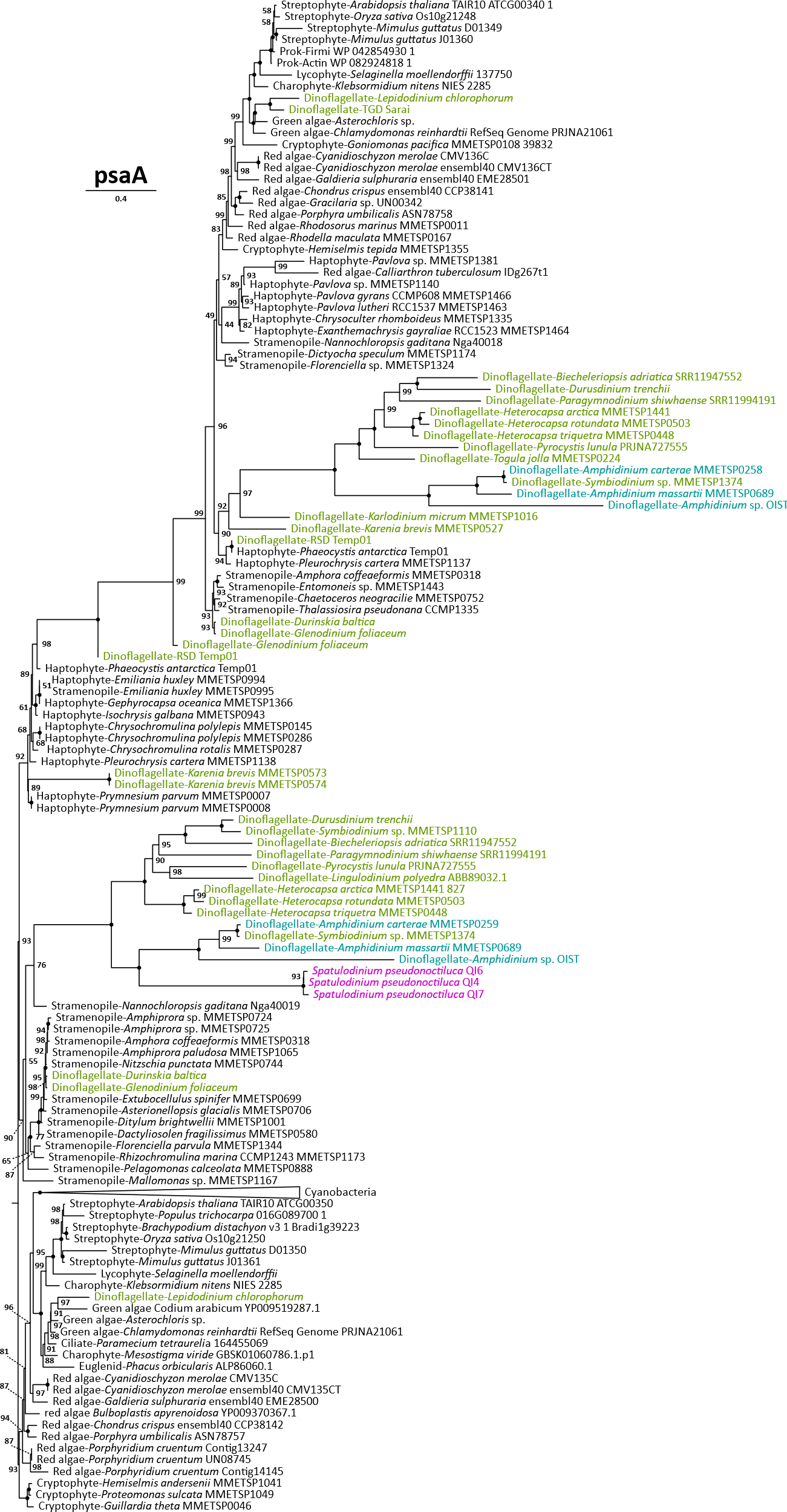

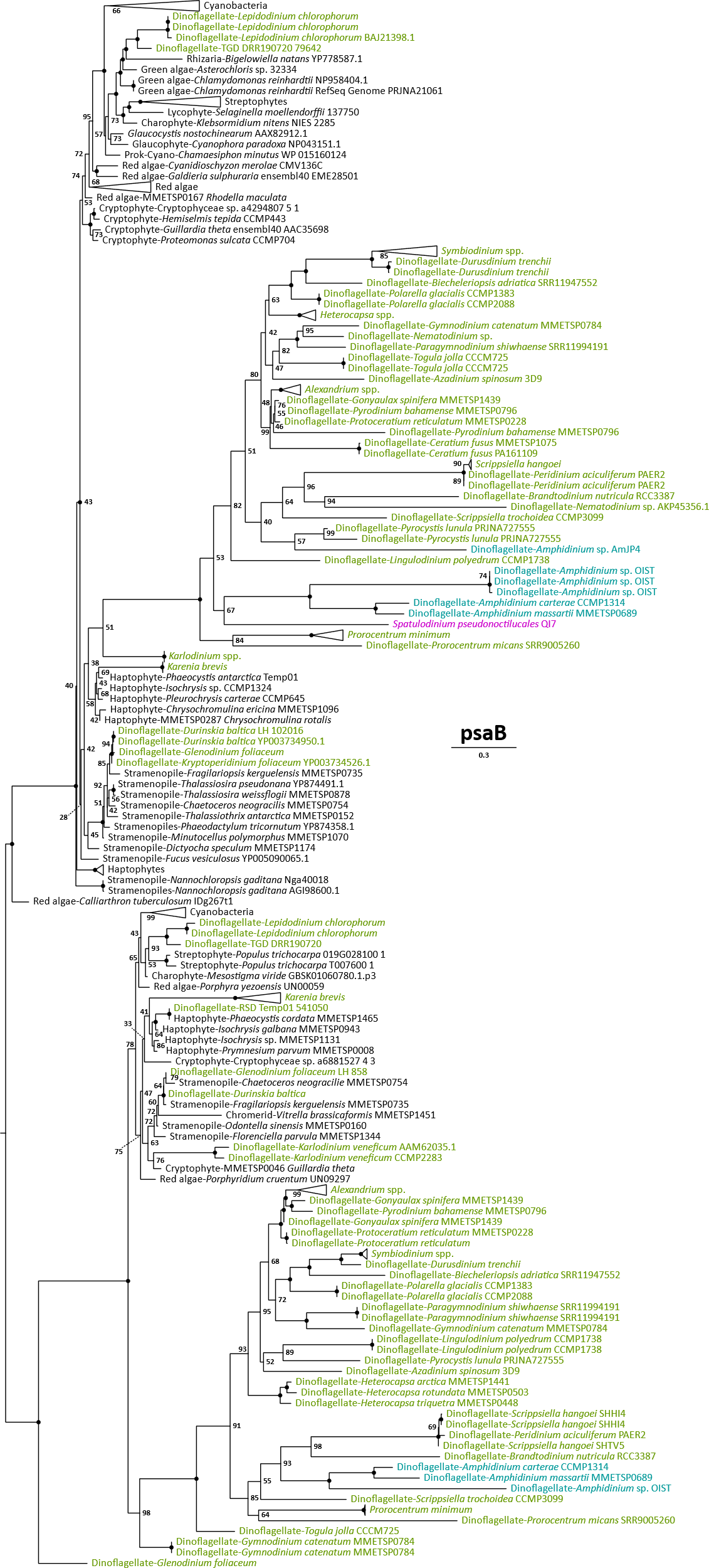

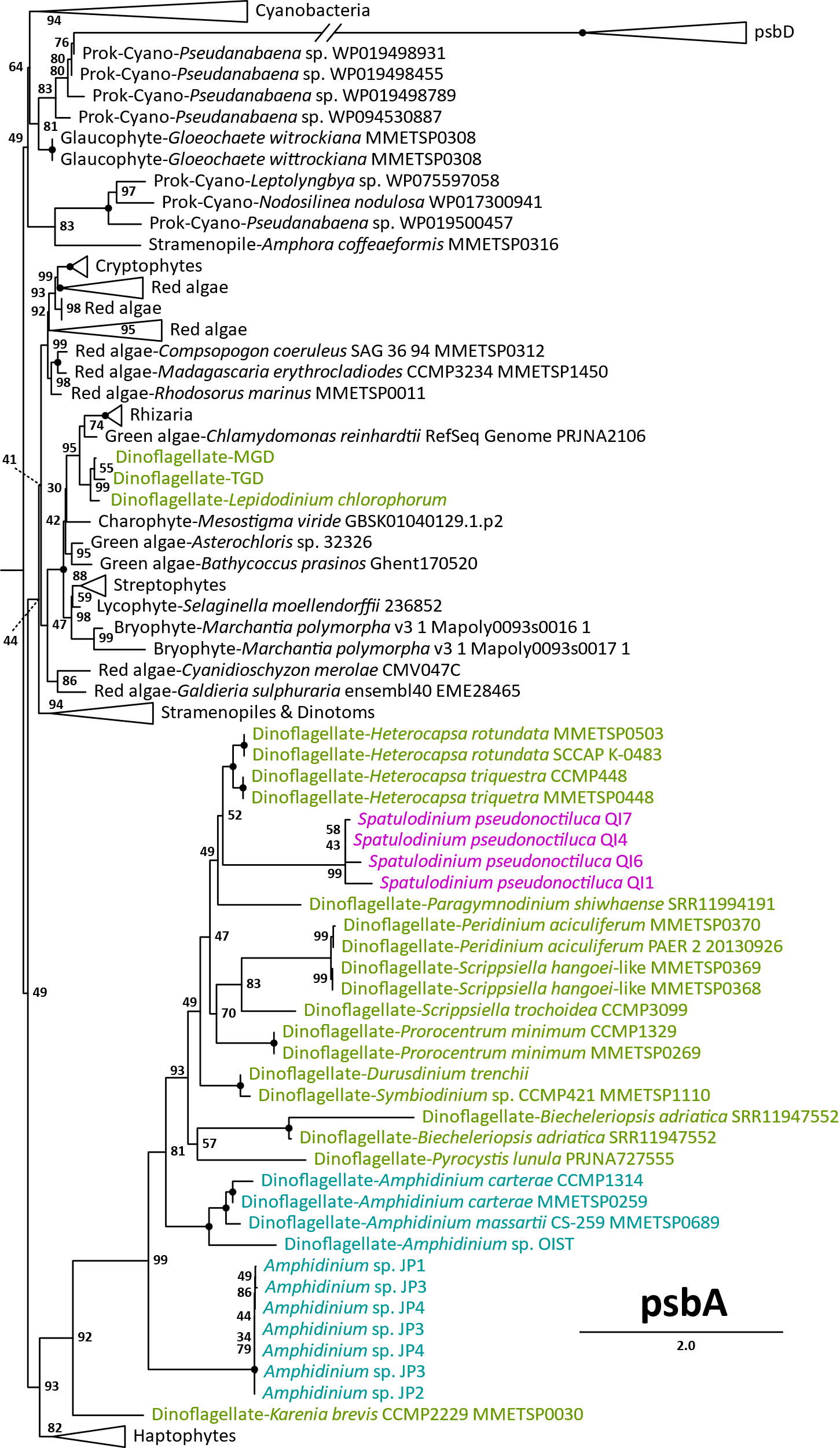

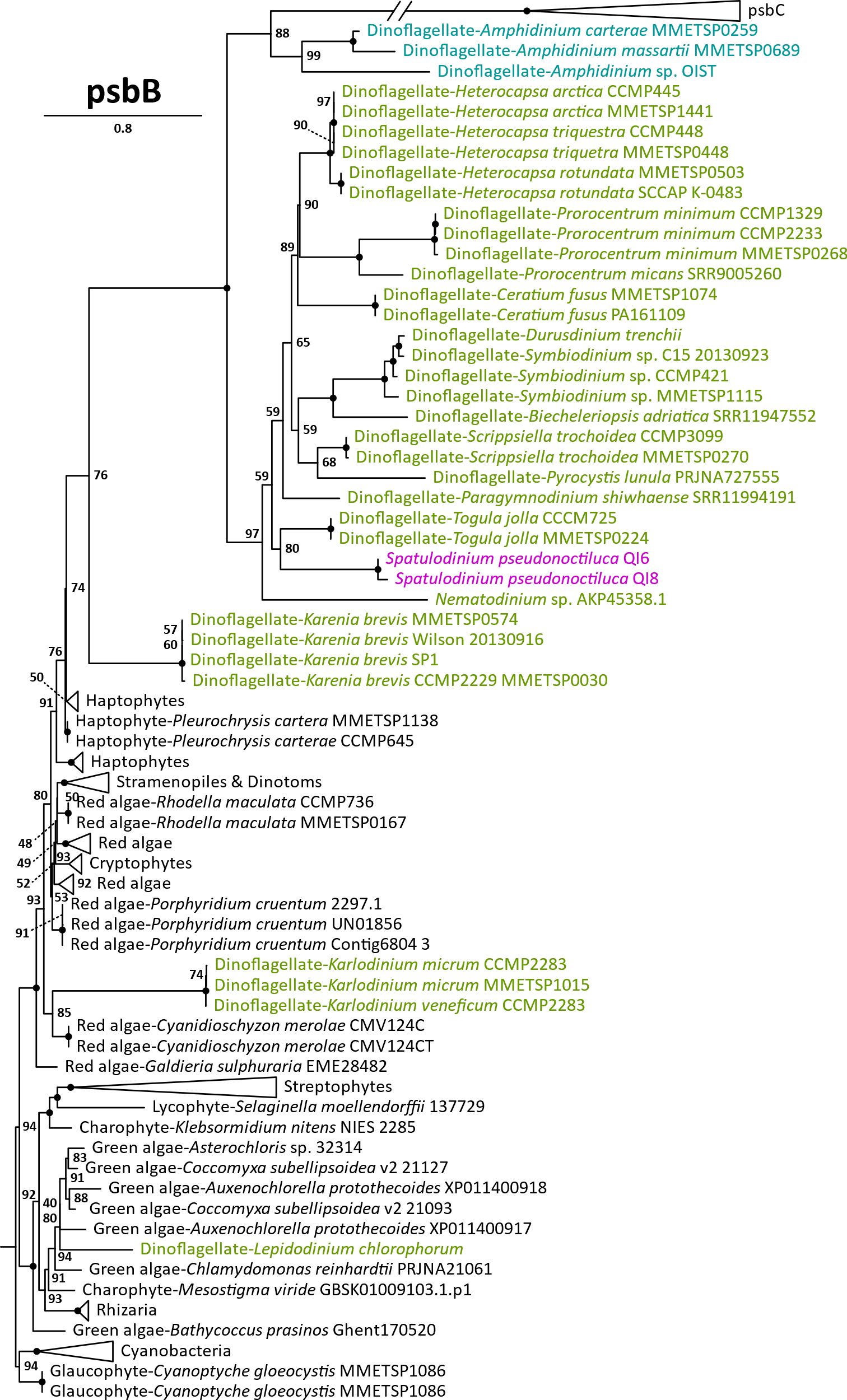

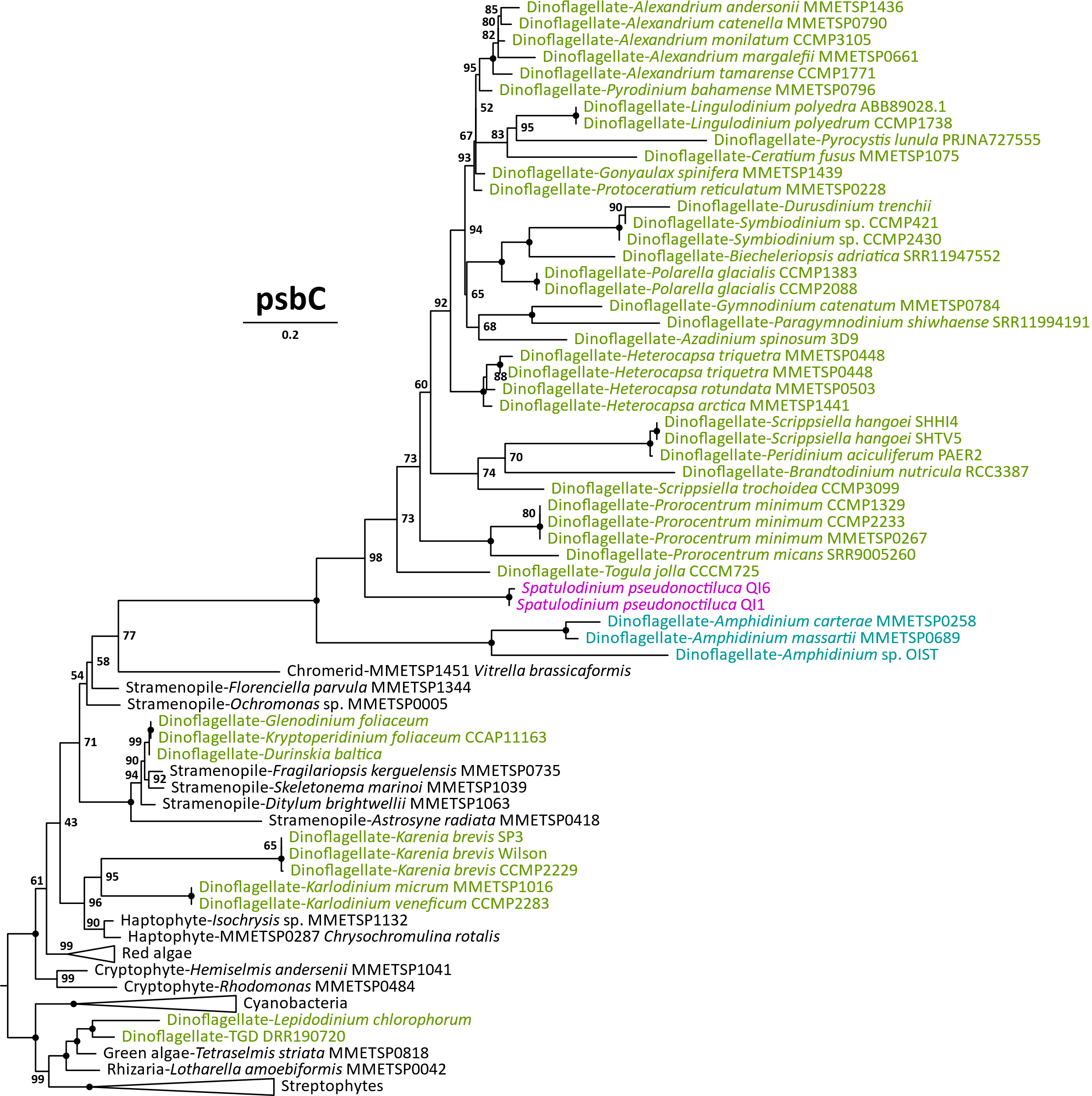
**

**Video captions (videos can be found at** [**https://doi.org/10.5281/zenodo.6326522**](https://doi.org/10.5281/zenodo.6326522)**):**

**Supplementary video S1. Unidentified cell (Ab-JP) belonging to the Abediniales lineage.**

**Supplementary video S2. Three *Fabadinium amicum* cells (Fa-JP1, Fa-JP2, and Fa-FC3).**

In order of appearance, arrows indicate the transverse flagellum of Fa-JP1, the transverse flagellum of Fa-FC3, and the longitudinal flagellum of Fa-FC3 as it is cast out.

**Supplementary video S3. Eight *Kofoidinium* sp. cells (Ko-JP1, Ko-FC2, Ko-QI3, Ko-QI4, Ko-QI5, Ko-QI6, Ko-QI7, and Ko-QI8).**

In order of appearance, arrows indicate a trailing thread-like flagellum of Ko-JP1, the transverse flagellum of Ko-FC2, the transverse flagellum of Ko-QI3, a long fiber observed near Ko-QI3, a long fiber observed near Ko-QI5, the longitudinal flagellum of Ko-QI5, and the longitudinal flagellum of Ko-QI6.

**Supplementary video S4. Eight *Spatulodinium pseudonoctiluca* cells (Sp-QI1, Sp-QI2, Sp-QI3, Sp-QI4, Sp-QI5, Sp-QI6, Sp-QI7, Sp-QI8).**

In order of appearance, arrows indicate the longitudinal flagellum of Sp-QI1, the transverse flagellum of Sp-QI1, the longitudinal flagellum of Sp-QI2, the transverse flagellum of Sp-QI2, the longitudinal flagellum of Sp-QI4, the transverse flagellum of Sp-QI4, a flagellum of Sp-QI5, the longitudinal flagellum of Sp-QI7, the transverse flagellum of Sp-QI7, the transverse flagellum of Sp-QI8, and the longitudinal flagellum of Sp-QI8.

**Supplementary video S5. Four unidentified *Amphidinium* sp. cells (Am-JP1, Am-JP2, Am-JP3, Am-JP4).**

**References**

Kishino H, Hasegawa M. 1989. Evaluation of the maximum likelihood estimate of the evolutionary tree topologies from DNA sequence data, and the branching order in Hominoidea. J. Mol. Evol. 29:170–179.

Kishino H, Miyata T, Hasegawa M. 1990. Maximum likelihood inference of protein phylogeny and the origin of chloroplasts. J. Mol. Evol. 31:151–160. doi: 10.1007/BF02109483.

Shimodaira H. 2002. An approximately unbiased test of phylogenetic tree selection. Syst. Biol. 51:492–508. doi: 10.1080/10635150290069913.

Shimodaira H, Hasegawa M. 1999. Multiple comparisons of log-likelihoods with applications to phylogenetic inference. Mol. Biol. Evol. 16:1114–1116. doi: 10.1093/oxfordjournals.molbev.a026201.

Strimmer K, Rambaut A. 2001. Inferring confidence sets of possibly misspecified gene trees. Proc. R. Soc. B Biol. Sci. 269:137–142. doi: 10.1098/rspb.2001.1862.

**Benthic database sources**

Birrer, S. C., Dafforn, K. A., Simpson, S. L., Kelaher, B. P., Potts, J., Scanes, P., & Johnston, E. L. (2018). Interactive effects of multiple stressors revealed by sequencing total (DNA) and active (RNA) components of experimental sediment microbial communities. Science of the Total Environment, 637:1383-1394.

Chaib De Mares, M., Sipkema, D., Huang, S., Bunk, B., Overmann, J., & Van Elsas, J. D. (2017). Host specificity for bacterial, archaeal and fungal communities determined for high-and low-microbial abundance sponge species in two genera. Frontiers in Microbiology, 8:2560.

Heger, T. J., Giesbrecht, I. J., Gustavsen, J., Del Campo, J., Kellogg, C. T., Hoffman, K.M., Lertzman, K., Mohn, W.W., & Keeling, P. J. (2018). High‐throughput environmental sequencing reveals high diversity of litter and moss associated protist communities along a gradient of drainage and tree productivity. Environmental Microbiology, 20(3):1185-1203.

Holman, L. E., de Bruyn, M., Creer, S., Carvalho, G., Robidart, J., & Rius, M. (2019). Detection of introduced and resident marine species using environmental DNA metabarcoding of sediment and water. Scientific reports, 9(1):1-10.

Little, M., George, E. E., Arts, M. G., Shivak, J., Benler, S., Huckeba, J., ... & Roach, T. N. (2021). Three-dimensional molecular cartography of the caribbean reef-building coral Orbicella faveolata. Frontiers in Marine Science, 8:627724.

Marcelino, V. R., & Verbruggen, H. (2016). Multi-marker metabarcoding of coral skeletons reveals a rich microbiome and diverse evolutionary origins of endolithic algae. Scientific Reports, 6(1):1-9.

Massana, R., Gobet, A., Audic, S., Bass, D., Bittner, L., Boutte, C., ... & De Vargas, C. (2015). Marine protist diversity in European coastal waters and sediments as revealed by high‐throughput sequencing. Environmental microbiology, 17(10):4035-4049.

Moreno-Pino, M., Cristi, A., Gillooly, J. F., & Trefault, N. (2020). Characterizing the microbiomes of Antarctic sponges: a functional metagenomic approach. Scientific reports, 10(1):1-12.

Nascimento, F. J., Lallias, D., Bik, H. M., & Creer, S. (2018). Sample size effects on the assessment of eukaryotic diversity and community structure in aquatic sediments using high-throughput sequencing. Scientific reports, 8(1):1-12.

Obiol, A., Giner, C. R., Sánchez, P., Duarte, C. M., Acinas, S. G., & Massana, R. (2020). A metagenomic assessment of microbial eukaryotic diversity in the global ocean. Molecular Ecology Resources, 20(3):718-731.

Pearman, J. K., Anlauf, H., Irigoien, X., & Carvalho, S. (2016). Please mind the gap – Visual census and cryptic biodiversity assessment at central Red Sea coral reefs. Marine Environmental Research, 118:20-30.

Reñé, A., Auladell, A., Reboul, G., Moreira, D., & López‐García, P. (2020). Performance of the melting seawater‐ice elution method on the metabarcoding characterization of benthic protist communities. Environmental Microbiology Reports, 12(3):314-323.

Rodríguez-Marconi, S., De la Iglesia, R., Díez, B., Fonseca, C. A., Hajdu, E., & Trefault, N. (2015). Characterization of bacterial, archaeal and eukaryote symbionts from Antarctic sponges reveals a high diversity at a three-domain level and a particular signature for this ecosystem. PloS one, 10(9):e0138837.

Rojas-Jimenez, K., Grossart, H. P., Cordes, E., & Cortés, J. (2020). Fungal communities in sediments along a depth gradient in the Eastern Tropical Pacific. Frontiers in microbiology, 11:575207.

Šlapeta, J., & Linares, M. C. (2013). Combined amplicon pyrosequencing assays reveal presence of the apicomplexan “type-N” (cf. Gemmocystis cylindrus) and Chromera velia on the Great Barrier Reef, Australia. PLoS One, 8(9):e76095.

Wang, Y., Tian, R. M., Gao, Z. M., Bougouffa, S., & Qian, P. Y. (2014). Optimal eukaryotic 18S and universal 16S/18S ribosomal RNA primers and their application in a study of symbiosis. PloS one, 9(3):e90053.

Wang, Y., Zhang, W. P., Cao, H. L., Shek, C. S., Tian, R. M., Wong, Y. H., ... & Qian, P. Y. (2014). Diversity and distribution of eukaryotic microbes in and around a brine pool adjacent to the Thuwal cold seeps in the Red Sea. Frontiers in microbiology, 5:37.

Wilms, R., Sass, H., Köpke, B., Köster, J., Cypionka, H., & Engelen, B. (2006). Specific bacterial, archaeal, and eukaryotic communities in tidal-flat sediments along a vertical profile of several meters. Applied and Environmental Microbiology, 72(4):2756-2764.

Xie, Y., Hong, S., Kim, S., Zhang, X., Yang, J., Giesy, J. P., ... & Khim, J. S. (2017). Ecogenomic responses of benthic communities under multiple stressors along the marine and adjacent riverine areas of northern Bohai Sea, China. Chemosphere, 172:166-174.

Zhang, W., Pan, Y., Yang, J., Chen, H., Holohan, B., Vaudrey, J., ... & McManus, G. B. (2018). The diversity and biogeography of abundant and rare intertidal marine microeukaryotes explained by environment and dispersal limitation. Environmental microbiology, 20(2):462-476.

SRA studies:

SRP013103: Unexpected diverse symbionts revealed by deep sequencing of sponge microbiome

SRP099130: Investigating the microbial eukaryotic communities in the surface waters of the Arafura sea and Coral Sea
